# 24-hour ambulatory blood pressure and cryptogenic ischemic stroke in young adults

**DOI:** 10.1080/07853890.2023.2203513

**Published:** 2023-04-22

**Authors:** Lauri Tulkki, Nicolas Martinez-Majander, Petri Haapalahti, Heli Tolppanen, Juha Sinisalo, Olli Repo, Tomi Sarkanen, Heikki Numminen, Essi Ryödi, Pauli Ylikotila, Risto O. Roine, Riikka Lautamäki, Antti Saraste, Tuuli Miettinen, Jaana Autere, Pekka Jäkälä, Marja Hedman, Juha Huhtakangas, Ulla Junttola, Jukka Putaala, Jani Pirinen, The SECRETO Study Group

**Affiliations:** a Department of Neurology, Helsinki University Hospital, Helsinki, Finland; b University of Helsinki, Helsinki, Finland; c HUS Diagnostic Center, Helsinki University Hospital, Helsinki, Finland; d Heart and Lung Center, Helsinki University Hospital, Helsinki, Finland; e Tampere University, Tampere, Finland; f Department of Neuroscience and Rehabilitation, Tampere University Hospital, Tampere, Finland; g Tays Heart Hospital, Tampere University Hospital, Tampere, Finland; h Neurocenter, Turku University Hospital, Turku, Finland; i University of Turku, Turku, Finland; j Heart Centre, Turku University Hospital, Turku, Finland; k Department of Neurology, Kuopio University Hospital, Kuopio, Finland; l University of Eastern Finland, Kuopio, Finland; m Heart Center, Kuopio University Hospital, Kuopio, Finland; n Department of Neurology, Oulu University Hospital, Oulu, Finland

**Keywords:** Cryptogenic ischemic stroke, young adults, ambulatory blood pressure monitoring, case–control study

## Abstract

**Background:**

In young patients, up to 40% of ischemic strokes remain cryptogenic despite modern-day diagnostic work-up. There are limited data on blood pressure (BP) behavior in these patients. Thus, we aimed to compare ambulatory blood pressure (ABP) profiles between young patients with a recent cryptogenic ischemic stroke (CIS) and stroke-free controls.

**Patients and Methods:**

In this substudy of the international multicenter case–control study SECRETO (NCT01934725), 24-hour ambulatory blood pressure monitoring (ABPM) was performed in consecutive 18–49-year-old CIS patients and stroke-free controls. The inclusion criteria were met by 132 patients (median age, 41.9 years; 56.1% males) and 106 controls (41.9 years; 56.6% males). We assessed not only 24-hour, daytime, and nighttime ABP but also hypertension phenotypes and nocturnal dipping status.

**Results:**

24-hour and daytime ABP were higher among controls. After adjusting for relevant confounders, a non-dipping pattern of diastolic blood pressure (DBP) was associated with CIS in the entire sample (odds ratio, 3.85; 95% confidence interval, 1.20–12.42), in participants without antihypertensives (4.86; 1.07–22.02), and in participants without a patent foramen ovale (PFO) (7.37; 1.47–36.81). After excluding patients in the first tertile of the delay between the stroke and ABPM, a non-dipping pattern of DBP was not associated with CIS, but a non-dipping pattern of both systolic BP and DBP was (4.85; 1.37–17.10). In participants with a PFO and in those without hypertension by any definition, no associations between non-dipping patterns of BP and CIS emerged.

**Conclusions:**

Non-dipping patterns of BP were associated with CIS in the absence of a PFO but not in the absence of hypertension. This may reflect differing pathophysiology underlying CIS in patients with versus without a PFO. Due to limitations of the study, results regarding absolute ABP levels should be interpreted with caution.

**Key Messages**
- Nocturnal non-dipping patterns of blood pressure were associated with cryptogenic ischemic stroke except in participants with a patent foramen ovale and in those without hypertension by any definition, which may indicate differing pathophysiology underlying cryptogenic ischemic stroke in patients with and without a patent foramen ovale.
- It might be reasonable to include ambulatory blood pressure monitoring in the diagnostic work-up for young patients with ischemic stroke to detect not only the absolute ambulatory blood pressure levels but also their blood pressure behavior.

## Introduction

The worldwide incidence of ischemic stroke (IS) in young adults has been rising, and currently, up to more than two million adults aged <50 years are estimated to suffer an IS each year [1]. Careful diagnostic work-up to define the most likely IS etiology is paramount to guide secondary prevention and to inform patients and their relatives on the nature and prognosis of the disease. However, despite comprehensive modern-day diagnostic tests, up to about 40% of IS cases in young adults remain without a known cause or with conditions where causality is difficult to prove conclusively, such as a patent foramen ovale (PFO) [2,3]. These strokes are traditionally labeled as cryptogenic, and the younger the patient group, the higher is the frequency of cryptogenic ischemic stroke (CIS) [2,3].

Hypertension is the most important risk factor for IS in general [4] and among the top two risk factors in young adults [5,6]. However, hypertension may be overlooked in clinical practice if diagnoses are based only on incidental daytime office or home blood pressure (BP) measurements. Importantly, ambulatory blood pressure monitoring (ABPM) is not performed routinely in IS patients, which might lead to further missed hypertension diagnoses. ABPM can identify masked and nocturnal hypertension and nocturnal non-dipping patterns of BP (i.e. BP decreases at night less than what is considered normal) [7]. Masked hypertension is associated with a first-ever stroke [8]. Furthermore, raised nocturnal BP is associated with a higher frequency of cardiovascular events, including stroke, but results regarding associations between non-dipping patterns of BP and stroke risk have been inconsistent [9–11].

There are limited data on ambulatory blood pressure (ABP) profiles in young IS patients. In the Norwegian Stroke in the Young Study (NOR-SYS), ABPM was performed in 15–60-year-old IS patients. In one substudy of NOR-SYS, the prevalence rates of a non-dipping pattern of BP and raised nocturnal BP were 38% and 51%, respectively [12]. In another substudy of NOR-SYS, the prevalence of masked hypertension was 12% [13]. In a third substudy of NOR-SYS, >40% of the CIS patients discharged with antihypertensive treatment had uncontrolled hypertension three months after the index stroke [14]. In the first two of those studies, the results were not reported separately for different IS subtypes [12,13].

To the best of our knowledge, there are no studies investigating ABP patterns specifically in young CIS patients. Thus, we aimed to compare ABP profiles between them and stroke-free controls. We hypothesized that compared to stroke-free controls, young CIS patients would have higher ABP levels and be more likely to exhibit masked hypertension and non-dipping patterns of BP.

## Patients and methods

### Study population

In the international prospective multicenter case–control study SECRETO (Searching for Explanations for Cryptogenic Stroke in the Young: Revealing the Etiology, Triggers, and Outcome; registration: www.clinicaltrials.gov, NCT01934725), 18–49-year-old patients hospitalized due to the first-ever imaging-positive CIS were included after a standardized, timely diagnostic work-up and examined according to the standardized protocol described in more detail previously [15]. The following diagnostic tests were mandatory for the inclusion of patients: brain magnetic resonance imaging, routine blood tests, screening for common thrombophilia, 12-lead electrocardiography, at least 24 h of Holter monitoring or continuous in-hospital electrocardiography monitoring with automated atrial fibrillation detection, imaging of cervicocephalic arteries, and standardized transthoracic and transesophageal echocardiography (a performance protocol for echocardiography has been described separately [16]). After these procedures, a patient was classified according to the A-S-C-O classification by the highest level of diagnostic evidence, and if the grade was 0 (absence of disease), 2 (causality uncertain), or 3 (unlikely a direct cause) in all four phenotypes [17], an IS was labeled as cryptogenic, and the patient could be included. However, as an adaptation to A-S-C-O, we included all patients with a PFO to ensure inclusion of a complete spectrum of PFO-related strokes. Stroke severity was evaluated with NIH Stroke Scale on admission.

In the main study, age- (±5 years), sex-, and ethnicity-matched stroke-free community controls were searched locally at each study center, sources including random search through population registers where possible, and included in a 1:1 fashion. However, we chose to conduct this substudy in unmatched format to prevent ABPM data loss and to increase the number of subjects as every patient and control did not have a pair with valid ABPM data for different reasons. For example, at the time of this study, a control subject had not yet been recruited for each patient, in some case–control pairs, ABPM was unsuccessful in either the patient or the control, and not every subject was willing to have ABPM performed (e.g. due to the long distance between home and hospital). Of the consecutive patients and controls enrolled between October 2015 and February 2020 in the university hospitals of Helsinki, Kuopio, Tampere, and Turku, we included all subjects in whom ABPM was performed successfully, which resulted in different numbers of patients, controls, and case–control pairs.

For the present study, a PFO was diagnosed by transcranial Doppler ultrasound with bubble study [18] and/or by echocardiography in both patients and controls. None of the patients underwent PFO closure prior to the ABPM.

The study was approved by the Ethics Committee of the Helsinki and Uusimaa Hospital District (362/13/03/00/2012) and local ethics committees at each recruiting center. A signed written informed consent from each participant, their legal representative, or next of kin was required for participation.

### Cardiovascular risk factors and comorbidities

A comprehensive clinical history was obtained from each participant by using a structured interview at a study visit and by reviewing medical records. The level of education was divided into two categories: high education (post-secondary non-tertiary education or higher) and low education (lower than post-secondary non-tertiary education). Physical activity of a usual week during the year before the stroke was assessed with the short, self-administered format of the International Physical Activity Questionnaire [19]. Physical inactivity was defined as the total metabolic equivalent of task minutes per week being less than the first tertile of the total metabolic equivalent of task minutes per week of the controls. A modified version of the Mediterranean Diet Score was used to evaluate participants’ diets, with a higher score indicating a healthier diet [20]. Obesity was defined as a waist-to-hip ratio of >0.85 in females and >0.90 in males [21]. Alcohol use was assessed with an adapted version of the World Health Organization’s Alcohol, Smoking and Substance Involvement Screening Test [22]. Heavy alcohol use was defined for females as using alcohol at least an average of two times per month and at least an average of five doses per time or more than seven doses per week, and for males as using alcohol at least an average of two times per month and at least an average of seven doses per time or more than 14 doses per week. Current tobacco smoking was defined as having smoked at least one cigarette during the year before the stroke. A history of hypertension was defined as having a prior hypertension diagnosis (excluding those diagnosed only when pregnant), being on antihypertensive medication at the time of stroke (in control subjects, at the time of their study visit), or having a mean office BP of ≥140/90 mm Hg on two measurements at a baseline study visit (in control subjects, at their study visit). Other registered comorbidities were hypercholesterolemia (a prior hypercholesterolemia diagnosis or antilipemic medication at the time of stroke), diabetes mellitus (a prior diagnosis of any diabetes or antidiabetic medication at the time of stroke), cardiovascular disease (a history of coronary heart disease, myocardial infarction, congestive heart failure, peripheral arterial disease, or atrial fibrillation), and a history of venous thrombosis.

### Office and ambulatory blood pressure measurements

Office BP measurements at study visits were mainly taken in a supine position. However, measurements in a sitting position were also accepted since the differences between supine and sitting BP are relatively small and since there is a large random variability in separate BP measurements regardless of body position [23]. In the present study, 24-hour ABPM was performed in consecutive patients and controls using existing validated equipment by Spacelabs Healthcare (Snoqualmie, WA, USA), Novacor (Rueil-Malmaison, France), and Schiller AG (Baar, Switzerland). In patients, ABPM was mainly performed close to the time of a baseline study visit or during a hospital stay; the vast majority of ABPMs in patients were performed after hospital discharge during normal daily activities except that most patients were on sick leave. ABP was mainly measured at 20-minute intervals during daytime and at 30-minute intervals during nighttime and in any body position participants were during measurements. Nighttime was set based on participants’ own estimations of bedtime and wake-up time or by default from 10:00 p.m. to 6:00 a.m. Quality criteria for ABPM were met if at least 70% of the BP measurements were valid and there were at least 20 valid daytime and seven valid nighttime BP measurements [7]. Participants were asked whether they slept poorly, moderately, or well during ABPM (referred to later as the quality of sleep). Other characteristics of ABPM are presented in their own table.

Hypertension phenotypes based on ABPM were categorized as follows: (a) 24-hour hypertension: a mean BP of ≥130/80 mm Hg during the whole monitoring; (b) daytime hypertension: a mean BP of ≥135/85 mm Hg during daytime; (c) nocturnal hypertension: a mean BP of ≥120/70 mm Hg during nighttime; (d) isolated nocturnal hypertension: fulfilling the criteria for nocturnal but not for 24-hour or daytime hypertension; (e) masked hypertension: fulfilling any criteria for hypertension on ABPM, not having a history of hypertension, and not being on antihypertensive medication during ABPM; (f) masked uncontrolled hypertension: fulfilling any criteria for hypertension on ABPM, a mean office BP of <140/90 mm Hg at a baseline study visit (in control subjects, at their study visit), and being on antihypertensive medication during ABPM; and (g) uncontrolled hypertension: fulfilling any criteria for hypertension on ABPM and being on antihypertensive medication during ABPM [7]. Additionally, hypertension by any definition was defined as fulfilling the criteria for a history of hypertension and/or any criteria for hypertension on ABPM. Regarding these definitions, worth noting is that some participants were on antihypertensive medication during ABPM although they did not fulfil the criteria for hypertension by any definition and that our definition of masked hypertension differs from the commonly used definition; participants who had a mean office BP of <140/90 mm Hg at a study visit and fulfilled any criteria for hypertension on ABPM but reported in a study interview that they had a prior hypertension diagnosis, even if they were not on antihypertensive medication, were not considered to have masked hypertension.

Participants were further classified into subgroups according to night-to-day ratios of systolic blood pressure (SBP) and diastolic blood pressure (DBP) as follows: extreme dippers (if a ratio was <0.80), normal dippers (≥0.80 to <0.90), decreased dippers (≥0.90 to <1.00), and reverse dippers (≥1.00) [9,10]. Since there were only few participants in the SBP reverse and extreme dipping categories, the reverse and decreased dippers were further grouped as non-dippers, and the normal and extreme dippers as dippers. DBP dipping categories were classified in the same manner to keep analyses consistent.

Furthermore, patients’ office BP was measured again at 3-month study visits. Hypertensive values at that time were defined as having a mean office BP of ≥140/90 mm Hg on two measurements. Uncontrolled hypertension at three months was defined as having hypertensive values despite being on antihypertensive medication at the time of a 3-month study visit.

### Statistics

Categorical variables were reported as n (%) and compared between groups with the Pearson chi-square or Fisher’s exact test. Continuous variables were reported as median with interquartile range (IQR) (and regarding NIH Stroke Scale score and antihypertensive medication, also with range because of its informative value for them) and compared between groups with the Mann–Whitney *U* test.

Binary logistic regression was used to assess the association of non-dipping patterns of BP (as dichotomous variables) and a dip in BP from daytime to nighttime (as continuous variables) with CIS in the entire study population and in different subgroups: (a) in all participants except patients in whom antihypertensive medication was initiated between the stroke and ABPM; (b) in participants without antihypertensives; (c) in all participants except patients in the first tertile of the delay between the stroke and ABPM; (d) in participants without hypertension by any definition; (e) in participants with hypertension by any definition; (f) in participants with a PFO; (g) in participants without a PFO; (h) in all participants with a PFO except patients in whom antihypertensive medication was initiated between the stroke and ABPM; (i) in all participants without a PFO except patients in whom antihypertensive medication was initiated between the stroke and ABPM; (j) in all participants with a PFO except patients in the first tertile of the delay between the stroke and ABPM; and (k) in all participants without a PFO except patients in the first tertile of the delay between the stroke and ABPM. Results were reported as odds ratios (OR) and 95% confidence intervals. All logistic regression models were adjusted for relevant confounders, which are mentioned separately for each model. Collinearity was tested using variance inflation factors.

Missing values of the physical activity questionnaire were imputed by using the worst scenario responses. For the diet score, each missing value was replaced by the mean value of available responses to that item, separately per patients and controls. We chose two different methods for imputing due to the different nature of these questionnaires; the diet questionnaire used was a multiple-choice questionnaire, whereas in the physical activity questionnaire, answers were given in numbers (days, hours, and minutes). If all responses to a questionnaire were missing, the final variable was coded as missing. Regarding these and all other missing values, we reported the number of participants with missing data and excluded them from the multivariable models.

We also performed further analyses to evaluate the possible effect of the delay between the stroke and ABPM on our results. In addition to the binary logistic regression analyses performed after excluding patients in the first tertile of the delay between the stroke and ABPM, selected characteristics, BP levels (also the office BP levels at 3-month study visits), and different hypertension and non-dipping phenotypes were compared between patients in the first tertile and those in the last two tertiles of the delay.

Statistical analyses were performed using IBM SPSS Statistics for Windows, version 25.0, and for Macintosh, version 28.0 (IBM Corp., Armonk, NY, USA). A *P*-value of <0.05 was considered significant.

## Results

### Characteristics of patients and controls

In total, 146 consecutive young CIS patients and 115 stroke-free controls were enrolled. Nine patients (6.2%) and two controls (1.7%) were excluded due to difficulties in ABPM or data handling, and five patients (3.4%) and seven controls (6.1%) due to insufficient quality of ABPM. Thus, 132 CIS patients (90.4%; median age, 41.9 years; IQR, 35.3–46.7; 56.1% males) and 106 stroke-free controls (92.2%; median age, 41.9 years; IQR, 34.4–46.8; 56.6% males) were included in the analysis. All subjects were white Europeans. There were 86 case–control pairs where ABPM was performed successfully in both the patient and the control. Forty-nine controls (46.2%) were recruited through the Finnish Population Register and the rest through other sources. In patients, the median NIH Stroke Scale score on admission was one (IQR, 0–3; range, 0–13) and the median delay between the stroke and ABPM was 12 days (IQR, 7–28). Compared to controls, patients were more frequently current tobacco smokers and had a poorer diet and more frequently a PFO. Other comorbidities were rare overall, but 35.6% of the patients and 28.3% of the controls had a history of hypertension (Table 1).

**Table 1.** Characteristics of patients and controls included in the study.

Characteristic (no. of participants with missing data)	Patients (*n* = 132)	Controls (*n* = 106)	*P*
Age at the time of ABPM, y	41.9 (35.3–46.7)	41.9 (34.4–46.8)	0.913
Male sex	74 (56.1)	60 (56.6)	0.933
Low level of education (1)	64 (48.5)	40 (38.1)	0.109
Physical inactivity (4)	49 (38.0)	35 (33.3)	0.461
Diet score (4)	25 (22–29)	26 (24–30)	0.023
Obesity	72 (54.5)	47 (44.3)	0.118
Heavy alcohol use	18 (13.6)	7 (6.6)	0.079
Current tobacco smoking (1)	39 (29.8)	19 (17.9)	0.035
History of hypertension	47 (35.6)	30 (28.3)	0.231
Hypercholesterolemia	8 (6.1)	7 (6.6)	0.864
Diabetes mellitus	5 (3.8)	1 (0.9)	0.230
Cardiovascular disease	1 (0.8)	0	1.000
History of venous thrombosis	5 (3.8)	1 (0.9)	0.230
Patent foramen ovale (5)	76 (57.6)	28 (27.7)	<0.001

Data are median (interquartile range) or *n* (%). ABPM: ambulatory blood pressure monitoring.

### Blood pressure levels and non-dipping patterns of blood pressure

A new antihypertensive treatment was initiated after the stroke in 37 patients (28.0%) resulting in 54 patients (40.9%) being on antihypertensive treatment during ABPM. Of the controls, nine (8.5%) were using antihypertensive medication during ABPM. Other characteristics of ABPM are presented in Table 2.

**Table 2.** Characteristics of ambulatory blood pressure monitoring.

Characteristic (no. of participants with missing data)	Patients (*n* = 132)	Controls (*n* = 106)	*P*
Duration, h	23.7 (22.8–24.0)	23.7 (23.2–24.3)	0.224
Percentage of successful measurements	95.2 (90.5–98.0)	92.0 (85.8–96.0)	<0.001
Successful measurements during			
the whole monitoring	60 (55–64)	60 (56–64)	0.679
daytime	43 (39–48)	44 (40–48)	0.156
nighttime	16 (14–18)	16 (14–17)	0.046
Length of nighttime, h	8.0 (8.0–9.0)	8.0 (7.0–8.7)	<0.001
Participants with poor quality of sleep during monitoring (26)	36 (29.8)	28 (30.8)	0.873
Participants on antihypertensive medication	54 (40.9)	9 (8.5)	<0.001
Number of antihypertensives per participant (in those on antihypertensive medication)	1 (1–1), 1–3	1 (1–1), 1–1	0.121
Frequency of taking antihypertensives (in those on antihypertensive medication)	1 (1–1), 1–4	1 (1–1), 1–1	0.102

Data are median (interquartile range); *n* (%); or median (interquartile range), range.

Except during nighttime, ABP levels were higher among controls compared to patients, even after excluding participants on antihypertensives. Patients’ office BP readings measured at baseline study visits did not significantly differ from controls’ office BP readings, but after excluding participants on antihypertensives, patients exhibited both lower office SBP and lower office DBP compared to controls (Table 3).

**Table 3.** Comparison of blood pressure levels between patients and controls.

	All participants (*n* = 238)			Participants without antihypertensives (*n* = 175)		
BP, mm Hg	Patients (*n* = 132)	Controls (*n* = 106)	*P*	Patients (*n* = 78)	Controls (*n* = 97)	*P*
Office SBP at a baseline study visit	121 (115–132)	125 (117–135)	0.126	119 (112–128)	125 (116–134)	0.004
Office DBP at a baseline study visit	74 (68–84)	76 (68–83)	0.725	72 (66–79)	76 (68–83)	0.045
Ambulatory						
24-hour SBP	117 (110–127)	122 (115–129)	0.014	115 (109–125)	121 (114–128)	0.004
24-hour DBP	75 (70–80)	77 (72–82)	0.016	72 (68–77)	77 (72–82)	0.002
daytime SBP	123 (115–132)	128 (120–134)	0.009	121 (114–129)	127 (119–133)	0.004
daytime DBP	79 (73–84)	82 (77–87)	0.002	77 (72–83)	81 (77–86)	<0.001
nighttime SBP	105 (98–116)	107 (99–114)	0.592	104 (96-110)	105 (99–113)	0.184
nighttime DBP	63 (59–72)	63 (59-69)	0.589	62 (57–68)	62 (58–68)	0.614

Data are median (interquartile range). BP: blood pressure; DBP: diastolic blood pressure; SBP: systolic blood pressure.

On ABPM, 37.1% of the patients and 46.2% of the controls fulfilled the criteria for at least one form of hypertension. Daytime hypertension was more frequent in controls compared to patients, also after excluding participants on antihypertensives. Masked hypertension was also more frequent in controls compared to patients but not after excluding participants on antihypertensive medication. Hypertension by any definition was observed in 50.8% of the patients and 51.9% of the controls (Table 4). Of the patients and controls on antihypertensive medication, 50.0% and 88.9%, respectively, had uncontrolled hypertension, i.e. they fulfilled the criteria for at least one form of hypertension based on ABPM despite being on antihypertensive medication.

**Table 4.** Prevalence of different hypertension and non-dipping phenotypes in patients and controls.

	All participants (*n* = 238)			Participants without antihypertensives (*n* = 175)		
Phenotype	Patients (*n* = 132)	Controls (*n* = 106)	*P*	Patients (*n* = 78)	Controls (*n* = 97)	*P*
24-hour hypertension	40 (30.3)	42 (39.6)	0.133	19 (24.4)	34 (35.1)	0.126
Daytime hypertension	35 (26.5)	44 (41.5)	0.015	15 (19.2)	37 (38.1)	0.007
Nocturnal hypertension	41 (31.1)	28 (26.4)	0.432	18 (23.1)	21 (21.6)	0.822
Any of the above three	49 (37.1)	49 (46.2)	0.156	22 (28.2)	41 (42.3)	0.054
Isolated nocturnal hypertension	7 (5.3)	2 (1.9)	0.305	3 (3.8)	2 (2.1)	0.657
Masked hypertension	13 (9.8)	25 (23.6)	0.004	13 (16.7)	25 (25.8)	0.146
Masked or masked uncontrolled hypertension	26 (19.7)	30 (28.3)	0.120	13 (16.7)	25 (25.8)	0.146
Uncontrolled hypertension	27 (20.5)	8 (7.5)	0.005	N/A	N/A	N/A
Hypertension by any definition	67 (50.8)	55 (51.9)	0.862	26 (33.3)	46 (47.4)	0.060
Non-dipping pattern of						
SBP	37 (28.0)	17 (16.0)	0.028	16 (20.5)	16 (16.5)	0.494
DBP	25 (18.9)	5 (4.7)	0.001	10 (12.8)	3 (3.1)	0.015
SBP and/or DBP	43 (32.6)	18 (17.0)	0.006	20 (25.6)	16 (16.5)	0.137
both SBP and DBP	19 (14.4)	4 (3.8)	0.006	6 (7.7)	3 (3.1)	0.190

Data are *n* (%). DBP: diastolic blood pressure; N/A: not applicable; SBP: systolic blood pressure. Hypertension by any definition was defined as fulfilling the criteria for a history of hypertension and/or any criteria for hypertension on ambulatory blood pressure monitoring.

There were two patients and no controls in the SBP reverse dipping category and 15 patients and 17 controls in the SBP extreme dipping category. In the DBP reverse dipping category, there were three patients and no controls. In the DBP extreme dipping category, there were 47 patients and 64 controls. All non-dipping patterns of BP were more frequent in patients compared to controls, but after excluding participants on antihypertensives, only a non-dipping pattern of DBP remained more frequent in patients (Table 4). Between all participants with at least moderate quality of sleep and those with poor quality of sleep during ABPM, there were no significant differences in the prevalence of different non-dipping patterns. Non-dipping patterns of DBP and both SBP and DBP were more frequent in all participants with a history of hypertension compared to those without a history of hypertension (22.1% vs. 8.1%, *p* = 0.002; and 15.6% vs. 6.8%, *p* = 0.033; respectively). All non-dipping patterns of BP were more frequent in all participants with hypertension by any definition compared to those without hypertension by any definition (a non-dipping pattern of SBP: 28.7% vs. 16.4%, *p* = 0.023; a non-dipping pattern of DBP: 19.7% vs. 5.2%, *p* < 0.001; a non-dipping pattern of SBP and/or DBP: 33.6% vs. 17.2%, *p* = 0.004; and a non-dipping pattern of both SBP and DBP: 14.8% vs. 4.3%, *p* = 0.006).

### Multivariable analyses

In the entire study population, logistic regression adjusted for confounders showed an association between a non-dipping pattern of both SBP and DBP and CIS with an OR of 4.34, between a non-dipping pattern of DBP and CIS with an OR of 3.85, and between a non-dipping pattern of SBP and/or DBP and CIS with an OR of 2.43. After excluding patients in whom antihypertensive medication was initiated between the stroke and ABPM, non-dipping patterns of DBP, SBP and/or DBP, and both SBP and DBP remained associated with CIS with ORs of 4.63, 2.39, and 4.17, respectively. After excluding all participants on antihypertensive medication, only the association between a non-dipping pattern of DBP and CIS remained significant with an OR of 4.86. After excluding patients in the first tertile of the delay between the stroke and ABPM (i.e. patients with a delay of less than nine days), non-dipping patterns of SBP and/or DBP and both SBP and DBP remained associated with CIS with ORs of 2.21 and 4.85, respectively (Table 5).

**Table 5.** Odds ratios and 95% confidence intervals from multivariable binary logistic regression models on the associations of non-dipping patterns of blood pressure and a dip in blood pressure with cryptogenic ischemic stroke.

Non-dipping pattern of BP/dip in BP from daytime to nighttime	All participants (*n* = 233)*	All participants except patients in whom antihypertensive medication was initiated between the stroke and ABPM (*n* = 197)^†^	Participants without antihypertensives (*n* = 172)^‡^	All participants except patients in the first tertile of the delay between the stroke and ABPM (*n* = 194)^§^
Non-dipping pattern of				
SBP	1.37 (0.62–3.02)	1.20 (0.50–2.87)	0.87 (0.34–2.26)	1.87 (0.78–4.49)
DBP	3.85 (1.20–12.42)	4.63 (1.31–16.36)	4.86 (1.07–22.02)	2.16 (0.54–8.56)
SBP and/or DBP	2.43 (1.25–4.70)	2.39 (1.16–4.90)	1.74 (0.77–3.94)	2.21 (1.07–4.56)
both SBP and DBP	4.34 (1.37–13.72)	4.17 (1.22–14.29)	2.40 (0.53–10.93)	4.85 (1.37–17.10)
10 mm Hg dip in SBP	1.16 (0.59–2.28)	0.99 (0.46–2.14)	1.37 (0.59–3.20)	0.78 (0.37–1.62)
10 mm Hg dip in DBP	0.31 (0.13–0.70)	0.33 (0.13–0.86)	0.24 (0.09–0.68)	0.58 (0.24–1.38)
10% dip in SBP	1.43 (0.58–3.53)	1.13 (0.40–3.17)	1.82 (0.59–5.60)	0.93 (0.35–2.45)
10% dip in DBP	0.36 (0.17–0.73)	0.41 (0.18–0.94)	0.31 (0.13–0.77)	0.59 (0.27–1.27)

ABPM: ambulatory blood pressure monitoring; BP: blood pressure; DBP: diastolic blood pressure; SBP: systolic blood pressure. All models are adjusted for age, sex, education, physical inactivity, diet score, obesity, heavy alcohol use, current tobacco smoking, and a history of hypertension. The models of a non-dipping pattern of SBP and the models of a dip in SBP are further adjusted for the same variable of DBP, and vice versa. No. of participants with missing data: *5 (2.1%), ^†^4 (2.0%), ^‡^3 (1.7%), and ^§^3 (1.5%).

In the models which were similarly adjusted except that the confounder ‘a history of hypertension’ was replaced with ‘hypertension by any definition’, non-dipping patterns of BP were correspondingly associated with CIS with slightly different ORs. After excluding participants with hypertension by any definition, none of the non-dipping patterns of BP were associated with CIS, whereas after excluding participants without hypertension by any definition, non-dipping patterns of SBP and/or DBP and both SBP and DBP were associated with CIS with ORs of 4.23 and 5.26, respectively (Table 6).

**Table 6.** Odds ratios and 95% confidence intervals from multivariable binary logistic regression models on the associations of non-dipping patterns of blood pressure and a dip in blood pressure with cryptogenic ischemic stroke (as a difference to Table 5, the confounder ‘a history of hypertension’ was replaced with ‘hypertension by any definition’.

Non-dipping pattern of BP/dip in BP from daytime to nighttime	All participants (*n* = 233)*	All participants except patients in whom antihypertensive medication was initiated between the stroke and ABPM (*n* = 197)^†^	Participants without antihypertensives (*n* = 172)^‡^	All participants except patients in the first tertile of the delay between the stroke and ABPM (*n* = 194)^§^	Participants without hypertension by any definition (*n* = 114)^ǁ^	Participants with hypertension by any definition (*n* = 119)^#^
Non-dipping pattern of						
SBP	1.45 (0.66–3.22)	1.26 (0.52-3.04)	0.95 (0.36–2.48)	2.12 (0.87–5.16)	0.77 (0.21–2.79)	2.12 (0.71–6.33)
DBP	4.12 (1.29–13.20)	5.15 (1.47–17.99)	5.26 (1.17–23.64)	2.17 (0.55–8.56)	8.06 (0.66–98.71)	3.88 (0.97–15.55)
SBP and/or DBP	2.66 (1.36–5.19)	2.64 (1.27–5.49)	1.95 (0.85–4.46)	2.51 (1.20–5.27)	1.46 (0.48–4.44)	4.23 (1.68–10.61)
both SBP and DBP	4.75 (1.50–15.05)	4.71 (1.38–16.08)	2.70 (0.59–12.34)	5.29 (1.49–18.70)	5.67 (0.54–60.09)	5.26 (1.33–20.84)
10 mm Hg dip in SBP	1.14 (0.58–2.25)	0.99 (0.45–2.16)	1.37 (0.59–3.22)	0.73 (0.35–1.53)	1.70 (0.55–5.28)	0.93 (0.37–2.37)
10 mm Hg dip in DBP	0.31 (0.13–0.70)	0.32 (0.12–0.83)	0.24 (0.08–0.67)	0.61 (0.25–1.47)	0.18 (0.04–0.77)	0.36 (0.12–1.04)
10% dip in SBP	1.38 (0.56–3.42)	1.15 (0.41–3.26)	1.84 (0.59–5.73)	0.86 (0.32–2.30)	2.74 (0.64–11.73)	0.90 (0.24–3.35)
10% dip in DBP	0.34 (0.17–0.70)	0.37 (0.16–0.85)	0.28 (0.11–0.70)	0.58 (0.27–1.26)	0.21 (0.06–0.72)	0.41 (0.15–1.08)

ABPM: ambulatory blood pressure monitoring; BP: blood pressure; DBP: diastolic blood pressure; SBP: systolic blood pressure. All models are adjusted for age, sex, education, physical inactivity, diet score, obesity, heavy alcohol use, and current tobacco smoking. The models of columns two to five are further adjusted for hypertension by any definition. In addition to these adjustments, the models of a non-dipping pattern of SBP and the models of a dip in SBP are further adjusted for the same variable of DBP, and vice versa. No. of participants with missing data: *5 (2.1%), ^†^4 (2.0%), ^‡^3 (1.7%), ^§^3 (1.5%), **^ǁ^**2 (1.7%), and **^#^**3 (2.5%).

None of the models including solely participants with a PFO showed any significant association between non-dipping patterns of BP and CIS. In the models including only participants without a PFO, non-dipping patterns of DBP, SBP and/or DBP, and both SBP and DBP were associated with CIS (ORs of 7.37, 4.48, and 6.06, respectively), also after excluding patients in whom antihypertensive medication was initiated between the stroke and ABPM (ORs of 9.58, 3.94, and 4.76, respectively). After excluding patients in the first tertile of the delay between the stroke and ABPM from the models including only participants without a PFO, an association of a non-dipping pattern of DBP with CIS was lost, but non-dipping patterns of SBP and/or DBP and both SBP and DBP remained associated with CIS with ORs of 3.88 and 7.81, respectively (Table 7).

**Table 7.** Odds ratios and 95% confidence intervals from multivariable binary logistic regression models on the associations of non-dipping patterns of blood pressure and a dip in blood pressure with cryptogenic ischemic stroke stratified by the status of patent foramen ovale.

	All participants		All participants except patients in whom antihypertensive medication was initiated between the stroke and ABPM		All participants except patients in the first tertile of the delay between the stroke and ABPM	
Non-dipping pattern of BP/dip in BP from daytime to nighttime	PFO (*n* = 101)*	no PFO (*n* = 127)^†^	PFO (*n* = 80)^‡^	no PFO (*n* = 112)^§^	PFO (*n* = 83)^ǁ^	no PFO (*n* = 106)^#^
Non-dipping pattern of						
SBP	1.33 (0.34–5.24)	1.55 (0.49–4.95)	1.80 (0.41–7.97)	1.10 (0.31–3.97)	1.62 (0.37–6.97)	2.32 (0.63–8.59)
DBP	3.17 (0.31–32.19)	7.37 (1.47–36.81)	2.78 (0.24–32.68)	9.58 (1.69–54.37)	1.27 (0.09–18.38)	4.03 (0.59–27.62)
SBP and/or DBP	1.52 (0.48–4.81)	4.48 (1.71–11.70)	1.72 (0.49–6.02)	3.94 (1.40–11.14)	1.19 (0.34–4.13)	3.88 (1.28–11.77)
both SBP and DBP	N/A	6.06 (1.46–25.19)	N/A	4.76 (1.02–22.17)	N/A	7.81 (1.52–40.05)
10 mm Hg dip in SBP	0.96 (0.28–3.32)	1.60 (0.60–4.29)	0.95 (0.23–3.95)	1.40 (0.47–4.12)	0.86 (0.24–3.03)	0.74 (0.23–2.34)
10 mm Hg dip in DBP	0.37 (0.08–1.68)	0.15 (0.04–0.54)	0.23 (0.04–1.50)	0.19 (0.05–0.75)	0.56 (0.12–2.77)	0.42 (0.11–1.58)
10% dip in SBP	0.93 (0.20–4.30)	2.09 (0.53–8.26)	0.81 (0.14–4.63)	1.84 (0.40–8.51)	0.86 (0.18–4.14)	0.83 (0.18–3.95)
10% dip in DBP	0.49 (0.14–1.73)	0.19 (0.06–0.57)	0.41 (0.09–1.79)	0.23 (0.07–0.80)	0.67 (0.17–2.55)	0.45 (0.14–1.45)

ABPM: ambulatory blood pressure monitoring; BP: blood pressure; DBP: diastolic blood pressure; N/A: not applicable because there were no controls with both a PFO and a non-dipping pattern of both SBP and DBP; PFO: patent foramen ovale; SBP: systolic blood pressure. All models are adjusted for age, sex, education, physical inactivity, diet score, obesity, heavy alcohol use, current tobacco smoking, and a history of hypertension. The models of a non-dipping pattern of SBP and the models of a dip in SBP are further adjusted for the same variable of DBP, and vice versa. No. of participants with missing data: *3 (2.9%), ^†^2 (1.6%), ^‡^2 (2.4%), ^§^2 (1.8%), ^ǁ^2 (2.4%), and ^#^1 (0.9%). Additionally, 5 participants were excluded from all these models due to missing PFO status data.

As a continuous variable, a dip in DBP from daytime to nighttime reduced the likelihood of CIS in the entire study population, also after excluding patients in whom antihypertensive medication was initiated between the stroke and ABPM, and in participants without antihypertensives but not after excluding patients in the first tertile of the delay between the stroke and ABPM (Tables 5 and 6). Furthermore, a dip in DBP from daytime to nighttime reduced the likelihood of CIS in participants without but not in those with hypertension by any definition (Table 6). None of the models including solely participants with a PFO showed any association between a dip in BP from daytime to nighttime and CIS (Table 7).

We found no evidence of significant collinearity in our models: the highest value of the variance inflation factor was 4.35 in the models including both a dip in SBP and a dip in DBP, whereas in other models, the highest observed variance inflation factor was 1.80.

### Comparison of patients stratified by the delay between the stroke and ambulatory blood pressure monitoring

Patients in the first tertile compared to those in the last two tertiles of the delay between the stroke and ABPM were significantly older (44.0 years vs. 40.5 years, *p* = 0.004) and more frequently obese (68.3% vs. 48.4%, *p* = 0.033) and had more frequently a history of hypertension (53.7% vs. 27.5%, *p* = 0.004). BP readings measured at baseline study visits and all ABP levels were significantly higher among patients in the first tertile compared to those in the last two tertiles. Based on ABPM, all other hypertension phenotypes except masked or masked uncontrolled hypertension and uncontrolled hypertension were more frequent in patients in the first tertile. Only a non-dipping pattern of DBP was more frequent in patients in the first tertile of the delay, whereas there were no significant differences in the prevalence of other non-dipping patterns of BP between these groups. At 3-month study visits, BP readings were higher and hypertensive values were more frequent in patients in the first tertile. Between the groups stratified by the delay, the proportions of those using antihypertensive medication did not significantly differ at the time of ABPM nor at 3-month study visits (Table 8).

**Table 8.** Selected characteristics, blood pressure levels, and different hypertension and non-dipping phenotypes of patients stratified by the delay between the stroke and ambulatory blood pressure monitoring.

Characteristic/BP, mm Hg/phenotype (no. of participants with missing data)	Patients with a delay of <9 days (*n* = 41)	Patients with a delay of ≥9 days (*n* = 91)	*P*
Age at the time of ABPM, y	44.0 (38.7–48.3)	40.5 (34.0–45.6)	0.004
Male sex	21 (51.2)	53 (58.2)	0.452
Physical inactivity (3)	16 (40.0)	33 (37.1)	0.752
Obesity	28 (68.3)	44 (48.4)	0.033
Heavy alcohol use	5 (12.2)	13 (14.3)	0.746
Current tobacco smoking (1)	11 (27.5)	28 (30.8)	0.706
History of hypertension	22 (53.7)	25 (27.5)	0.004
Patent foramen ovale	19 (46.3)	57 (62.6)	0.080
NIH Stroke Scale score on admission (1)	2 (1–3), 0–13	1 (0–2), 0–12	0.087
SBP at a baseline study visit	125 (118–139)	121 (114-129)	0.032
DBP at a baseline study visit	79 (72–89)	73 (67–81)	0.008
Delay between the stroke and ABPM, d	4 (3–6)	18 (12–33)	<0.001
Antihypertensive medication initiated between the stroke and ABPM	9 (22.0)	28 (30.8)	0.297
Antihypertensive medication at the time of ABPM	14 (34.1)	40 (44.0)	0.289
Ambulatory			
24-hour SBP	124 (112–134)	115 (109–125)	0.006
24-hour DBP	77 (72–84)	72 (69–78)	0.007
Daytime SBP	127 (116–138)	121 (114–129)	0.011
Daytime DBP	80 (75–87)	78 (73–82)	0.044
Nighttime SBP	109 (103–125)	104 (96–110)	0.003
Nighttime DBP	70 (64–77)	62 (57–67)	<0.001
24-hour hypertension	18 (43.9)	22 (24.2)	0.022
Daytime hypertension	16 (39.0)	19 (20.9)	0.029
Nocturnal hypertension	23 (56.1)	18 (19.8)	<0.001
Any of the above three	24 (58.5)	25 (27.5)	<0.001
Isolated nocturnal hypertension	5 (12.2)	2 (2.2)	0.030
Masked or masked uncontrolled hypertension	11 (26.8)	15 (16.5)	0.167
Uncontrolled hypertension	10 (24.4)	17 (18.7)	0.452
Non-dipping pattern of			
SBP	10 (24.4)	27 (29.7)	0.532
DBP	14 (34.1)	11 (12.1)	0.003
SBP and/or DBP	16 (39.0)	27 (29.7)	0.289
Both SBP and DBP	8 (19.5)	11 (12.1)	0.261
Antihypertensive medication at the time of a 3-month study visit	19 (46.3)	38 (41.8)	0.623
SBP at a 3-month study visit (7)	127 (117–139)	119 (111–127)	0.002
DBP at a 3-month study visit (7)	77 (72–85)	72 (67–79)	<0.001
Hypertensive values at three months (7)	10 (25.0)	9 (10.6)	0.036
Uncontrolled hypertension at three months (7)	7 (17.5)	7 (8.2)	0.139

Data are median (interquartile range); *n* (%); or median (interquartile range), range. ABPM: ambulatory blood pressure monitoring; BP: blood pressure; DBP: diastolic blood pressure; SBP: systolic blood pressure.

## Discussion

The main finding of our study was that different non-dipping patterns of BP were associated with CIS with some variations in different subgroups. Interestingly, in analyses stratified by the PFO status and in those stratified by the hypertension status, we observed that only in participants without a PFO and only in those with hypertension by any definition, associations between non-dipping patterns of BP and CIS were present. Another notable finding was that controls exhibited higher 24-hour and daytime ABP levels compared to patients, also after excluding participants on antihypertensives. Finally, hypertension was remarkably common and frequently uncontrolled among both our young patients and controls based on ABPM.

Forty-seven patients (35.6%) had a history of hypertension. Masked hypertension was detected by ABPM in 13 patients (9.8%), which is in accordance with the earlier finding of 12% with masked hypertension in NOR-SYS [13]. The total prevalence of hypertension (i.e. hypertension by any definition) in our patients was 50.8%, which outnumbered observations (44.2% and 40.7%) of earlier studies including non-selected young patients. The most likely explanation for this difference is that ABPM was not performed in those studies [5,6]. It should be noted that the sum of the prevalence rates of a history of hypertension and masked hypertension does not match the prevalence of hypertension by any definition since the antihypertensive medication was initiated between the stroke and ABPM in several patients. The prevalence rates of a history of hypertension and hypertension by any definition did not differ between our patients and controls.

It is important to note that our results regarding the differences in the hypertension phenotypes and in ABP levels between patients and controls should be interpreted with caution due to potential biases related to antihypertensive treatment status. Indeed, a new antihypertensive treatment was initiated between the stroke and ABPM in 37 patients. Furthermore, a new antihypertensive medication was already initiated before baseline study visits in several patients, which might have resulted in lower office BP levels. It is also possible that some patients without actual hypertension might have been on antihypertensive medication during ABPM since during the acute or subacute phase of stroke, antihypertensive treatment might have been initiated with a low threshold. However, only 13 of 54 patients on antihypertensive medication did not fulfil the criteria for hypertension by any definition. Furthermore, there were only two patients and two controls who reported that they did not have a prior hypertension diagnosis and had a mean office BP of <140/90 mm Hg at a study visit but were on antihypertensive medication at the time of stroke or, in the case of controls, at the time of a study visit. These four participants were considered to have a history of hypertension even though they were taking antihypertensive medication, for example, for migraine prevention. However, one of them fulfilled the criteria for hypertension on ABPM.

Of our patients on antihypertensives, 50.0% had their hypertension uncontrolled based on ABPM, which slightly outnumbered the percentage (>40% in CIS patients) observed in NOR-SYS. However, in our study, ABPM was mainly performed relatively close to the time of stroke compared to the 3-month interval between the stroke and ABPM in NOR-SYS [14], which is likely to increase the prevalence of different hypertension phenotypes in our patients. Actually, we explored how that interval may have affected those prevalence rates and BP levels in our patient population; we found that all hypertension phenotypes except masked or masked uncontrolled hypertension and uncontrolled hypertension were more frequent and all ABP levels were higher among patients in the first tertile compared to those in the last two tertiles of the delay. However, also the office BP levels measured at study visits, and even those measured three months after the stroke, were markedly higher among patients in the first tertile compared to those in the last two tertiles, which indicates that there might be other explanations for these differences in BP levels and in the prevalence of different hypertension phenotypes in addition to the delay between the stroke and ABPM. For example, patients in the first tertile of the delay were significantly older and more frequently obese and had more frequently a history of hypertension compared to those in the last two tertiles. Furthermore, it has been shown that BP levels are elevated directly after hospital admission in young patients with IS, but BP decreases already in the first 24 h of hospitalization [24].

The first observations on the association between non-dipping BP and stroke were published in 1988 [25], although since then, results regarding this association have been inconsistent in prospective studies and meta-analyses [9–11]. When dipping has been categorized into four groups, also extreme dipping has emerged as a predictor of stroke, resulting in a J-shaped relationship between stroke incidence and dipping patterns with the highest incidences in extreme and reverse dippers [9]. However, a meta-analysis showed that the effect of extreme dipping is significantly influenced by antihypertensive treatment; treated patients exhibiting extreme dipping had borderline lower risk of total cardiovascular events than treated patients exhibiting normal dipping. In that meta-analysis, analyses were also performed with dipping status classified into two groups, dipping and non-dipping, and a non-dipping pattern of SBP was associated with stroke with a hazard ratio of 1.43 [11].

In our study, no associations between a non-dipping pattern of SBP and CIS were observed, whereas a non-dipping pattern of DBP was associated with CIS in several different logistic regression models. There are many possible explanations for these observations: Previous studies on dipping and non-dipping have mainly focused on SBP [9–11], whereas those addressing DBP are rare. Also, study populations of those studies have generally been markedly older than ours, with a mean age of participants being >50 years [9–11], and actually, the choice to use variables derived from SBP readings has been explained by that in adults aged >50 years, SBP is considered a more relevant risk factor than DBP [10]. Indeed, SBP has shown to predominate over DBP as a predictor of coronary heart disease in older individuals related to stiffening of large arteries with age, but in individuals aged <50 years, DBP has noted to be the strongest predictor of coronary heart disease risk compared to SBP and pulse pressure [26]. However, in a recent prospective cohort study including older adults (mean age, 70.8 years), ambulatory DBP predicted first-ever stroke more robustly than ambulatory SBP, and also office DBP was associated with first-ever stroke, whereas office SBP was not [27]. In addition to the non-dipping pattern of DBP, non-dipping patterns of SBP and/or DBP and both SBP and DBP were associated with CIS in many of our models, and of these two, the non-dipping pattern of both SBP and DBP more strongly. This observation might indicate that the more impaired the regulation of BP, the higher is the risk of CIS, or the CIS itself might be the cause of that impairment. Nevertheless, these observations strengthen the rationale for assessing the associations of both SBP and DBP dipping status with stroke risk.

We found also PFO status to modulate the associations between BP dipping status and stroke. In participants with a PFO, there were no associations between any non-dipping patterns of BP and CIS. However, in participants without a PFO, there were strong associations in several logistic regression models, which is likely to reflect differing pathophysiology underlying CIS in patients with versus without a PFO. In patients with PFO-related CIS, abnormalities in coagulation and fibrinolysis may act as the driving force promoting thrombosis in the venous system and lead to a stroke *via* paradoxical embolism [28], whereas in those without a PFO, strokes may be, at least partly, related to possible vascular changes linked to non-dipping BP. This theory is also supported by our observation that whether a participant had hypertension or not also seemed to affect the associations between BP dipping status and stroke; in the models including solely participants without hypertension by any definition, none of the non-dipping patterns of BP were associated with CIS, but in the models including solely those with hypertension by any definition, associations between non-dipping patterns of BP and CIS were observed. However, a dip in DBP reduced the likelihood of CIS in the models including only participants without hypertension by any definition. Moreover, the logistic regression models which were adjusted for hypertension by any definition showed associations between non-dipping patterns of BP and CIS. The two last-mentioned findings suggest that in young CIS patients, non-dipping BP may also play a role independent of hypertension status.

In addition to the issues related to antihypertensive treatment status and ABP levels, our study has other limitations. Although our aim was to enroll consecutive patients, the possibility of some selection exists; some patients with more severe symptoms might have been left out due to inability to consent and adhere to the protocol. It is also possible that controls who are willing to participate in studies like this might be more aware of their health than patients, resulting in that controls might also have taken better care of themselves and be healthier overall compared to patients. Neither the sleep time nor the timing of ABPM was fixed, the latter of which resulted in a variation in the delay between the stroke and ABPM. Nevertheless, non-dipping patterns of SBP and/or DBP and both SBP and DBP remained associated with CIS even after excluding patients in the first tertile of the delay. Furthermore, in patients in the first tertile, the median delay was four days (IQR, 3–6), whereas raised BP is known to decrease already in the first 24 h of hospitalization for IS [24]. ABPM performed closer to the time of stroke might also reflect pre-stroke circumstances better than later performed monitoring since patients who underwent ABPM sooner may not yet had taken actions to reduce BP and since antihypertensive medication may not yet had reached its full effect. On the other hand, in addition to the potential effect of a recent IS on BP, a recent IS might affect quality of sleep, which might further have an effect on nocturnal BP. Yet, compared to controls, our patients did not report more frequently poor quality of sleep during ABPM (Table 2).

The main strengths of our study were a standardized study protocol, where only patients with imaging-verified strokes were included after a timely diagnostic work-up, and a structured data collection with only a few missing data. Furthermore, only few participants were excluded from the original sample. We were able to assess most of the well-known major risk factors for IS in both patients and controls and to use those risk factors as confounders in logistic regression models. To consider other possible confounding factors, we performed the analyses also after excluding participants on antihypertensive medication and separately after excluding those patients in the first tertile of the delay between the stroke and ABPM from logistic regression models, and still non-dipping BP remained associated with CIS. Furthermore, we were uniquely able to consider the PFO status of both patients and controls in our analyses. Although case–control studies cannot prove causality, the new information provided by our study is valuable, especially given that longitudinal studies seem unfeasible in this study question because of the relatively low incidence of IS in young adults. It would necessitate performing ABPM in extensive number of young adults aged <50 years and following up those for several decades to observe sufficient number of cases of early-onset CIS to assess the associations between BP behavior and CIS.

## Conclusions

Several different non-dipping patterns of BP were significantly associated with CIS, even after adjusting for multiple confounders. Regarding the presence of a PFO, these associations applied only to those without a PFO, and regarding the presence of hypertension, they applied only to those with hypertension by any definition. These findings might reflect differing pathophysiology underlying CIS in patients with and without a PFO. Based on our findings, the non-dipping BP, specifically in young CIS patients in the absence of a PFO, may be a treatable risk factor. Interestingly, young CIS patients had lower 24-hour and daytime ABP levels compared to stroke-free controls. Also, the prevalence of hypertension by any definition did not differ between our patients and controls. This might indicate that hypertension alone is not as significant a risk factor for IS in young CIS patients as in general. However, due to limitations of the study, results regarding absolute ABP levels should be interpreted with caution. Finally, although causality cannot be inferred, our results should act as an incentive to perform ABPM in young CIS patients to detect not only the absolute ABP levels, but also how BP behaves in these patients.

## Data Availability

The corresponding author had full access to all the data in the study and takes responsibility for its integrity and the data analysis. The data of this study may be available from the corresponding author upon reasonable request.
